# Roles of Changes in Active Glutamine Transport in Brain Edema Development During Hepatic Encephalopathy: An Emerging Concept

**DOI:** 10.1007/s11064-013-1141-x

**Published:** 2013-09-26

**Authors:** Magdalena Zielińska, Mariusz Popek, Jan Albrecht

**Affiliations:** Department of Neurotoxicology, Mossakowski Medical Research Centre, Polish Academy of Sciences, Pawinskiego St. 5, 02-106 Warsaw, Poland

**Keywords:** Hepatic encephalopathy, Astrocytic swelling, Brain edema, Glutamine, Glutamine transporters, Blood–brain barrier

## Abstract

Excessive glutamine (Gln) synthesis in ammonia-overloaded astrocytes contributes to astrocytic swelling and brain edema, the major complication of hepatic encephalopathy (HE). Much of the newly formed Gln is believed to enter mitochondria, where it is recycled to ammonia, which causes mitochondrial dysfunction (a “Trojan horse” mode of action). A portion of Gln may increase osmotic pressure in astrocytes and the interstitial space, directly and independently contributing to brain tissue swelling. Here we discuss the possibility that altered functioning of Gln transport proteins located in the cellular or mitochondrial membranes, modulates the effects of increased Gln synthesis. Accumulation of excess Gln in mitochondria involves a carrier-mediated transport which is activated by ammonia. Studies on the expression of the cell membrane N-system transporters SN1 (SNAT3) and SN2 (SNAT5), which mediate Gln efflux from astrocytes rendered HE model-dependent effects. HE lowered the expression of SN1 at the RNA and protein level in the cerebral cortex (cc) in the thioacetamide (TAA) model of HE and the effect paralleled induction of cerebral cortical edema. Neither SN1 nor SN2 expression was affected by simple hyperammonemia, which produces no cc edema. TAA-induced HE is also associated with decreased expression of mRNA coding for the system A carriers SAT1 and SAT2, which stimulate Gln influx to neurons. Taken together, changes in the expression of Gln transporters during HE appear to favor retention of Gln in astrocytes and/or the interstitial space of the brain. HE may also affect arginine (Arg)/Gln exchange across the astrocytic cell membrane due to changes in the expression of the hybrid Arg/Gln transporter y^+^LAT2. Gln export from brain across the blood–brain barrier may be stimulated by HE via its increased exchange with peripheral tryptophan.

## Gln Accumulation as a Cause of Astrocytic Swelling and Brain Edema in HE: A Brief Overview

Hepatic encephalopathy (HE) is a complex neuropsychiatric syndrome caused by liver failure where excessive accumulation of blood-derived ammonia in the brain is a primary causative factor. Acute HE or aggravation of a chronic condition due to hyperammonemic incidents is associated with brain edema, which often leads to the patients’ death in consequence of increased intracranial pressure and herniation (reviewed in [1, 2]). Brain edema is primarily cytotoxic in nature and is mainly due to astrocytic swelling (AS [1]). The current view is that excessive synthesis of Gln from ammonia and glutamate catalyzed by an astrocyte-specific enzyme, glutamine synthetase (GS) plays a major role in the pathogenesis of AS. The natural history of this view has been exhaustively described in recent review articles [3, 4]. Briefly, the concept originates from the observation that Gln in cultured astrocytic increases oxidative stress in 6-diazo-5-oxo–l-norleucine (DON), histidine (His) and cyclosporine A-sensitive manner. It has also been shown that HE in experimental animals subside or became attenuated upon treatment with a GS inhibitor, methionine sulfoximine (MSO). The abnormalities in the brain corrected by MSO include, among other events, decreased oxygen consumption and large neutral amino acid imbalance [5], and, at the physiological level, decreased specific gravity and/or increased water content of the tissue, reflecting brain edema [6, 7]. At the cellular level, MSO reduces perivascular astrocytic and pericytic swelling in cerebral cortex (cc) of hyperammonemic rats [8, 9], and swelling of cultured astrocytes exposed to ammonia [10]. The toxic effects of Gln are believed to be largely due to its entry to mitochondria and subsequent intra-mitochondrial release of toxic concentrations of ammonia, which leads to mitochondrial permeability transition (mPT) and swelling [11]. This sequence of events has been derived from in vitro studies and has been summarized as the “Trojan horse” hypothesis [3]. According to this hypothesis, Gln acting as a “Trojan horse” would contribute to other actions of ammonia at different cellular targets, collectively resulting in the complex interplay of oxidative/nitrosative stress and impairment of in- and out-transport of different osmolytes, leading to intracellular osmotic imbalance [12, 13]. However, the Trojan horse hypothesis still needs to be unequivocally validated in the in vivo setting. One of the weak points of the hypothesis is related to the controversy whether glutamine is located in the inner mitochondrial membrane or in the inter-membrane space as suggested by Kvamme et al. [14]. Independently, accumulation of Gln is believed to directly contribute to the osmotic imbalance in astrocytes [15].

While part of newly accumulated Gln may passively diffuse from the locus of its synthesis, its considerable fraction is directed towards different destinations by active transport mediated by specific carriers. The present paper addresses the as yet unresolved question whether and in what degree modulation of the carriers by the pathogenic condition affects the Gln-related pathogenesis. In the following section we provide basic information about how Gln is shuttled between the cells and subcellular compartments of the brain and how it manages to egress the brain to the periphery.

## Gln Transport in the CNS

### Mitochondrial Gln Transport

Cerebral mitochondria possess an active, saturable transport system for Gln. The system operates at relatively high affinity and low capacity and is therefore well suited for the regulation of the entry of Gln from cytoplasm, where it is present at low milimolar concentrations [16]. Mitochondrial Gln uptake is inhibited by several neutral amino acids, of which histidine (His) appears to exert the strongest inhibitory effect [17].

### Gln Transport in Astrocytes and Neurons

Gln transporting proteins are present both in astrocytes and neurons, but are distributed between them asymmetrically. Astrocytes are enriched in two bi-directional system N transporters: SN1 [18] and SN2 [19]. SN1 is the best candidate to specifically mediate Gln efflux from astrocytes. SN1 has an activity optimum at physiological extracellular Gln concentration (Km ~0.4 mM) and shows independence on a substrate on the trans-side, which predisposes it to be a mediator of net efflux [18]. Furthermore, SN1 activity is positively controlled by intracellular Glu [20], and the significance of this interdependence is underscored by the fact that neuron-derived neurotransmitter Glu accounts for ~80–90 % of the substrate pool for Gln synthesis in astrocytes [21]. SN2 is less ubiquitous in the CNS than SN1 and in addition to Gln release, mediates glycine release for regulation of the *N*-methyl-d-aspartate (NMDA) receptor function [22]. Still, the precise role of each of the two transporters in regulating Gln efflux from astrocytes and its distribution in the different compartments of the CNS is not completely clear as yet. In neurons, Gln uses as carriers two neuronal system A transporters: SAT1 and SAT2, of which SAT2 specifically serves to replenish Glu in glutamatergic neurons [23]. Part of Gln migrates between the different cellular compartment in exchange for arginine (Arg), using the y^+^L system. The y^+^L system is represented by 4F2hc/y^+^LAT1 and 4F2hc/y^+^LAT2 transporters, which in addition to cationic amino acids accept neutral amino acids; the transport of the latter is coupled to Na^+^ [24]. Of these two transporters, the brain expresses only y^+^LAT2 [25, 26], which appears to abound in astrocytes and as such is well suited for catalyzing Gln/Arg exchange in these cells [27].

### Gln Transport in the Blood–Brain Barrier-Forming Cerebral Capillary Endothelial Cells

Available evidence suggests that Gln transport at the blood–brain barrier (BBB) is primarily mediated by system N transporters [18, 28]. Studies with luminal and abluminal plasma membrane vesicles derived from bovine brain endothelial cells disclosed that system N transporters account for ~80 % of Gln transport from brain to peripheral blood [29], and the presence of SN1 in the endothelial cells has been well documented [30]. Gln out-transport from the brain is also facilitated by system L-mediated exchange with other large neutral amino acids, mainly tryptophan (Try) [31, 32]. The Arg/Gln exchanger y^+^LAT2 appear to be likewise expressed in the cerebral capillary endothelial cells in culture, but much less so in situ [33].

## Effects of HE on Gln Transport in the Brain and Expression of Transporters Residing in the Different Compartments: State of the Art

### Gln Uptake in Brain Slices

**Fig. 1:**
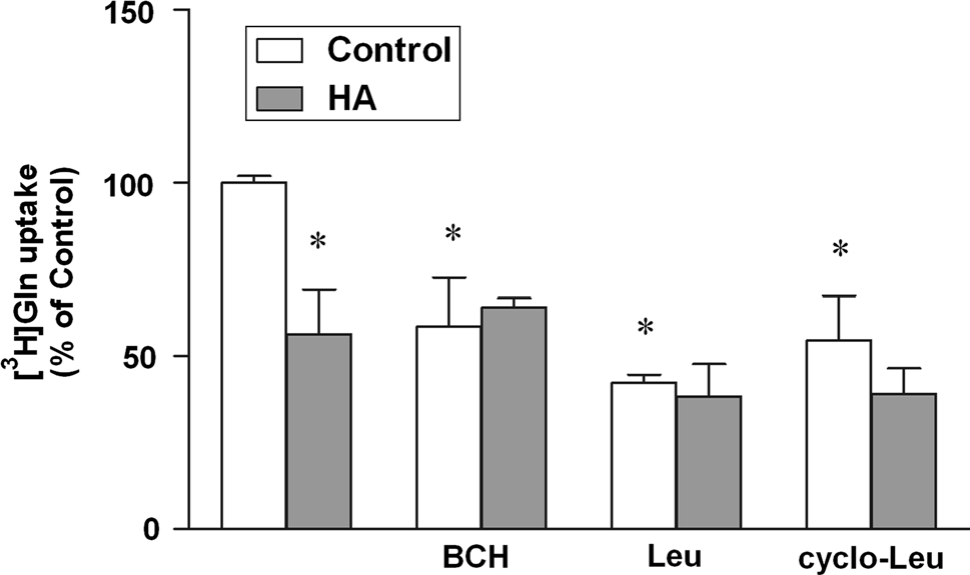
Effect of the presence of 10 mM BCH, Leu and *cyclo*-Leu on [^3^H] Gln uptake in control and hyperammonemic (HA) rat cerebral cortical slices. Cerebral cortical slices of HA rat were pre-incubated for 30 min at 37 °C and the uptake was started by adding L-[^3^H] Gln (Perkin-Elmer) at 100 mmol/l final concentration and the incubation was continued for 4 min. The incubation was terminated by rapid vacuum filtration, followed by three washes with 2 ml with Krebs buffer maintained at 4 °C. The radioactivity on filter disks was measured in a liquid scintillation spectrometer. The control value for [^3^H] Gln uptake was 29.5 nmol/min/mg wet tissues. Values in each group are mean ± SD for n = 5. (*p < 0.05; Dunnet’s test)

HA [3 i.p. injections of ammonium acetate (600 mg per kg) at 24 h intervals] decreased L-[^3^H] Gln uptake to the cerebral cortical slices, and the decrease affected the component of uptake sensitive to leucine (Leu), 2-aminobicyclo(2,2,1)heptane-2-carboxylic acid (BCH), and *cyclo*-leucine, indicating selective vulnerability to system L (Fig. 1).

### Mitochondria

Convincing evidence that transport of excess of newly formed Gln to brain mitochondria is carrier-mediated has come from studies analyzing the effects of the transport competitor, His. Co-incubation with His abolished Gln-evoked mPT and swelling, and the response was well correlated with inhibition of Gln uptake [34]. His inhibited swelling of ammonia-treated astrocytes and the effect showed cooperativity with MSO [35]. His administered i.p. attenuated brain edema and other oxidative stress-related responses in the brain in rats with thioacetamide (TAA)-induced HE [36]. However, results obtained with His must be interpreted with caution do to its pleiotropic effects, including direct amelioration of HE-induced oxidative stress, as manifested by the recovery of mitochondrial glutathione [37], and, independently of the tissue status, interference with system N-mediated Gln transport at the astrocytic cell membranes [38], and references therein). An earlier observation indicated that ammonia increases Gln uptake to non-synaptic (astrocytic) mitochondria [39], which is likely to augment the mitochondrial effects of excess Gln in the setting of HE, where brain ammonia is elevated as a rule [2, 32].

### Astrocytes and Neurons

**Fig. 2:**
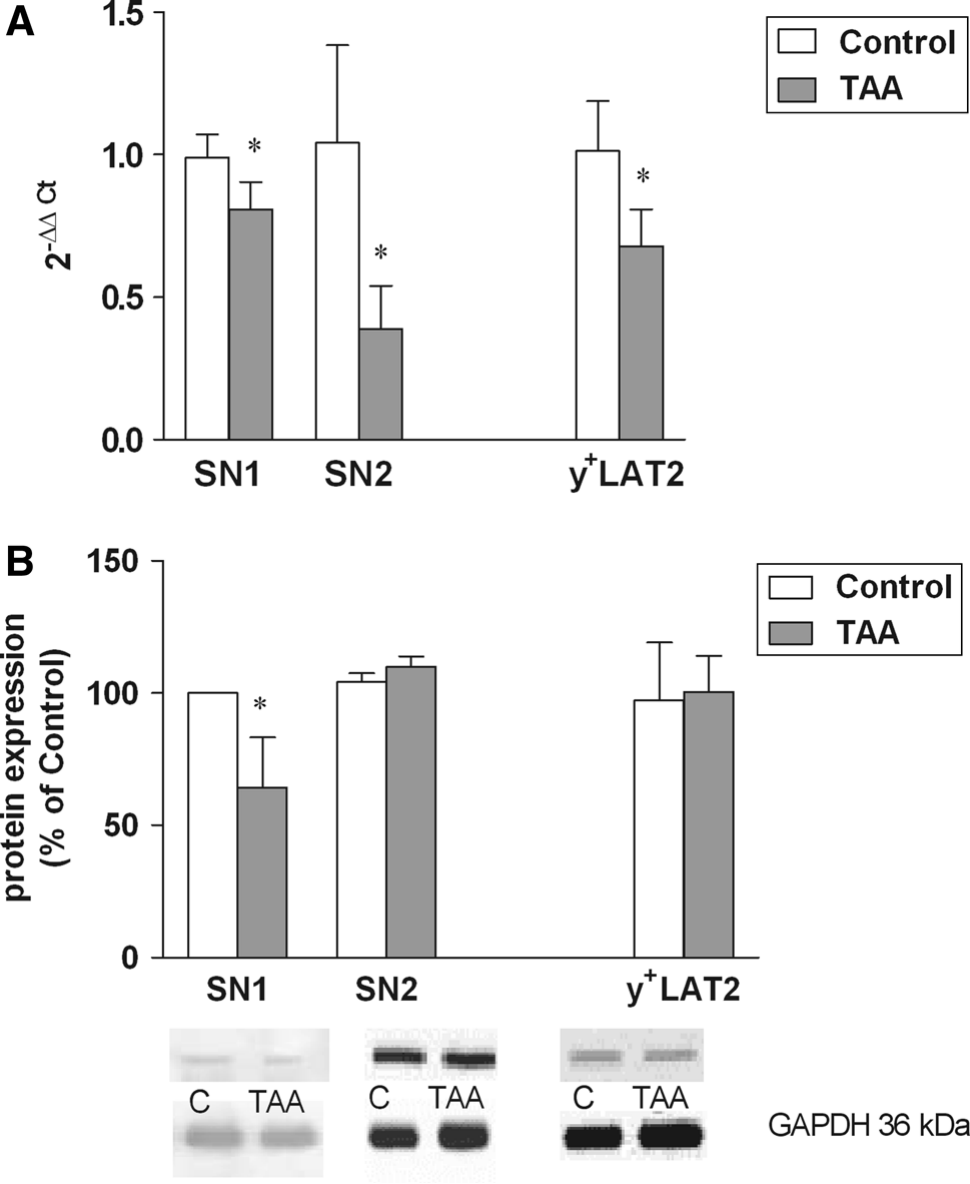
**a**, **b** Expression of SN1, SN2 and y^+^LAT2 at the mRNA (**a**) and protein (**b**) level in cerebral cortex of control rats and rats with TAA-induced HE. **a** Relative quantification of SN1, SN2, y^+^LAT2 mRNA. Total RNA was isolated using TRI Reagent (Sigma-Aldrich), and reverse-transcribed using High Capacity cDNA Reverse Transcribed Kit (Life Technologies; Applied Biosystems). Probes for SN1, SN2, y^+^LAT2 and β-actin (Rn 01447660, Rn 00684896, Rn 01431908_m1 and Rn 00667869, respectively) were purchased from Applied Biosystems. Further details were as described in Ref. [27]. Values in each group are mean ± SD for n = 8; *p < 0.05; *T* test. **b** Quantification of SN1, SN2, y^+^LAT2 protein densities. The antibodies used included SN1 (Santa Cruz Biotechnology; goat polyclonal, 1:500), SN2 (Santa Cruz Biotechnology; goat polyclonal, 1:1,000), y^+^LAT2 (Santa Cruz Biotechnology; 1:1,000, rabbit polyclonal) and GAPDH (Sigma-Aldrich; rabbit polyclonal, 1:3,000). Representative immunoblots of SN1, SN2, y^+^LAT2 and GAPDH (loading control) corresponding to the immunoblots of transporters. See Ref. [27] for further experimental details. Values in each group are mean ± SD for n = 5–8. (*p < 0.05; *T* test)

**Fig. 3:**
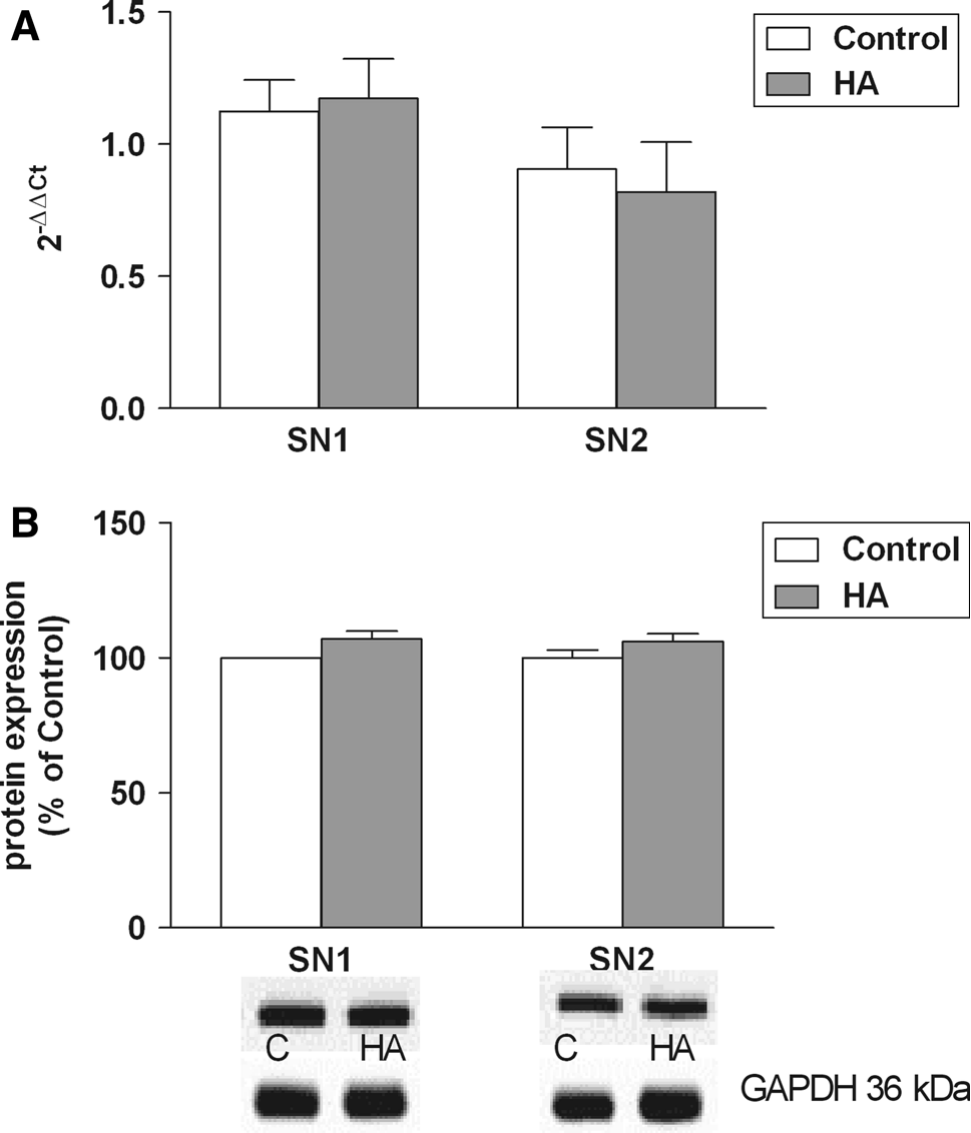
**a**, **b** Expression of SN1 and SN2 at the mRNA (**a**) and protein (**b**) level in cerebral cortex of control rats and rats with ammonium acetate-induced HA. Values in each group are mean ± SD for n = 4

Experiments carried out in this laboratory demonstrated a marked decrease of SN1 and SN2 expression at the mRNA and SN1 at the protein level, in the cc of rats with TAA-induced HE (Fig. 2a, b), coinciding with marked cc swelling in this model [40]. Interestingly, SN1 and SN2 expression remained unaltered in rats with simple hyperammonemia (HA) in the ammonium acetate model (Fig. 3a, b), where (cc) does not change its volume [40]. Two other groups have measured the effects of HE on SN2 mRNA expression in two different models. Our results are in agreement with decreased SN2 mRNA expression in the acute liver failure (azoxymethane) model in mice reported by Desjardins et al. [41]. The results of the present study are also compatible with the unchanged expression of SN2 protein in the mice TAA model similar to our model, as revealed immunocytochemically [42]. It must be reemphasized, however that, SN1 but not SN2 is believed to be the key mediator of the efflux of newly synthesized Gln from astrocytes [38].

A variable response of the y^+^LAT2 carrier has been noted in the two different models. Simple hyperammonemia (HA) stimulated the expression of y^+^LAT2 mRNA and protein in the brain, accounting for an increased back-flux of Gln to the cells (probably astrocytes) on the expense of Arg [27]. HE in the TAA model decreased the brain y^+^LAT2 expression at the mRNA level, leaving the y^+^LAT2 protein unchanged (Fig. 2a, b). The ambiguity of the responses noted, and the impact of the observed changes on the overall tissue balance of Gln remain to be clarified.

### Transport Across the BBB

The effect of HE or ammonia on the system N-mediated Gln transport in the cerebral capillary endothelial cells has not been examined as yet. The L system-dependent exchange of systemic Try with intracerebral Gln has been found increased in cerebral capillary endothelial cells treated with ammonia [31], or isolated from rats with HE [32]. While the results tend to support increased efflux of newly formed Gln from the brain to the periphery in the setting of HE, the significance of the observation cannot be appreciated before its role relative to the system N-mediated transport is unraveled. Ammonia also increased y^+^LAT2 expression in the cerebral capillary endothelial cell line [43], but how this increase translates into Gln/Arg exchange and whether, and in what degree, it is eventually reflected in the tissue distribution of Gln is unknown.

## Conclusions and Perspectives

Collectively, the above presented data suggest that HE-induced alterations in the expression of the Gln transporting proteins may favor Gln trapping within the vulnerable compartments of the brain. According to the most plausible scenario, decreased expression of the astrocytic Gln transporters SN1 (SNAT3) and SN2 (SNAT5), if translated into their decreased activity (this presumption remains to be tested), will impair Gln efflux from astrocytes. This in turn will lead to increased intra-astrocytic and in consequence, intra-mitochondrial accumulation of Gln, the latter process being additionally stimulated by ammonia-induced activation of the mitochondrial Gln carrier. This sequence of mutually amplifying events would contribute to astrocytic swelling both by the cytoplasmic—osmotic mechanism, and by the mitochondrial—Trojan horse model.

A question that remains to be resolved is how to reconcile the elevation of intraastrocytic Gln content with the increase of extracellular Gln, frequently observed in HE [15, 44, 45]. One possible explanation would be that the apparent increase of extracellular Gln concentration is due to the shrinkage of extracellular space subsequent to astrocytic swelling, as earlier suggested for the increased accumulation of extracellular K^+^ in the brains of hyperammonemic rats [46]; in such a case, however, the increase would encompass, in a nonselective way, other amino acids as well. One other possibility would be a decreased backflux of Gln to astrocytes, due to an unfavorable concentration gradient. Clearly, further work is needed to resolve between these two possibilities.

Consequences of the decreased expression of the neuronal transporters SAT1 and SAT2 are more difficult to predict. If a portion of Gln newly derived from astrocytes indeed “omits” neurons, this may either lead to increased Gln efflux across the BBB, or contribute to its increased trapping in the extracellular space. The latter interpretation would be consistent with the earlier discussed increase of extracellular Gln often recorded in human HE patients [15] and animal HE models [44, 45].

Clearly, interpretations of the results derived so far will have to be verified by detailed investigation on how the changes expression of the different Gln-transporting moieties are translated into their functional status. While preliminary data obtained in this laboratory demonstrate that ammonia, the key primary cause of astrocytic swelling does inhibit Gln uptake in vitro (Fig. 1), the inhibition appears to primarily involve the sodium-independent L system, which does not appear compatible with the transporter expression data. However, the complexity of cellular composition of the slice and the ensuing difficulty to analyze the contribution of the different cell components to the final result, do not permit simple extrapolation of the results obtained in vitro to the in vivo conditions. Assessment of the role of alterations in Gln transport in the redistribution of Gln overload between the different compartments of the CNS will not be possible without knowledge is acquired about quantitative contribution of active transport and diffusion to Gln fluxes. To this end, Gln fluxes will have to be compared in the brains of normal rats and rats with selectively inactivated transporters (knockout animals, siRNA), using the NMR technique.
